# Role of Lysozyme Inhibitors in the Virulence of Avian Pathogenic *Escherichia coli*

**DOI:** 10.1371/journal.pone.0045954

**Published:** 2012-09-26

**Authors:** Lise Vanderkelen, Ellen Ons, Joris M. Van Herreweghe, Lien Callewaert, Bruno M. Goddeeris, Chris W. Michiels

**Affiliations:** 1 Laboratory of Food Microbiology and Leuven Food Science and Nutrition Research Centre (LFoRCe), Katholieke Universiteit Leuven, Leuven, Belgium; 2 Laboratory for Vaccine Design, Katholieke Universiteit Leuven, Leuven, Belgium; Centre National de la Recherche Scientifique, Aix-Marseille Université, France

## Abstract

Lysozymes are key effectors of the animal innate immunity system that kill bacteria by hydrolyzing peptidoglycan, their major cell wall constituent. Recently, specific inhibitors of the three major lysozyme families occuring in the animal kingdom (c-, g- and i-type) have been discovered in Gram-negative bacteria, and it has been proposed that these may help bacteria to evade lysozyme mediated lysis during interaction with an animal host. *Escherichia coli* produces two inhibitors that are specific for c-type lysozyme (Ivy, Inhibitor of vertebrate lysozyme; MliC, membrane bound lysozyme inhibitor of c-type lysozyme), and one specific for g-type lysozyme (PliG, periplasmic lysozyme inhibitor of g-type lysozyme). Here, we investigated the role of these lysozyme inhibitors in virulence of Avian Pathogenic *E. coli* (APEC) using a serum resistance test and a subcutaneous chicken infection model. Knock-out of *mliC* caused a strong reduction in serum resistance and in *in vivo* virulence that could be fully restored by genetic complementation, whereas *ivy* and *pliG* could be knocked out without effect on serum resistance and virulence. This is the first *in vivo* evidence for the involvement of lysozyme inhibitors in bacterial virulence. Remarkably, the virulence of a *ivy mliC* double knock-out strain was restored to almost wild-type level, and this strain also had a substantial residual periplasmic lysozyme inhibitory activity that was higher than that of the single knock-out strains. This suggests the existence of an additional periplasmic lysozyme inhibitor in this strain, and indicates a regulatory interaction in the expression of the different inhibitors.

## Introduction

Lysozymes are key effectors of innate immunity in all animals (for review, see 2). They catalyze the hydrolysis of β-(1–4) glycosidic bonds between the N-acetylmuramic acid and N-acetylglucosamine repeating units composing the backbone of peptidoglycan, the major constituent of bacterial cell walls. Lysozyme is a component of both phagocytic and secretory granules of neutrophils and is also produced by monocytes, macrophages and epithelial cells. It is found in significant concentrations in saliva, airway mucus, milk and other secretions, and is considered to be an important first line barrier against bacterial infection. While many gram-positive bacteria are rapidly killed by lysozyme *in vitro*, gram-negative bacteria are not because they have an outer membrane that prevents direct access of lysozyme to the peptidoglycan sacculus. However, *in vivo*, gram-negative bacteria are sensitized to lysozyme by accessory antimicrobial peptides of the innate immunity system such as defensins and complement which disrupt the outer membrane barrier [1]. Several structurally different lysozymes have been described and the major types within the animal kingdom are the c-type (chicken or conventional type), the g-type (goose type) and the i-type (invertebrate type) lysozymes. Vertebrates have genes for both c- and g-type lysozyme, but their spatio-temporal expression is species-specific. The chicken genome for instance comprises a single c-type and two g-type lysozyme genes. The c-type gene is highly expressed in the oviduct under control of steroid hormones, as well as in macrophages, where expression is further enhanced by bacterial lipopolysaccharides [2]. In the intestine of young chickens, c-type lysozyme gene expression was observed up to an age of 8 days, while the g-type lysozyme genes, *g1* and *g2*, were expressed at all ages up to at least 38 days [3]. Further, g-type lysozyme was identified in the liver, kidney, bone marrow and lung tissue of chicken [3], [4].

In view of the widespread occurrence of lysozymes, it is not surprising that commensal and pathogenic bacteria colonizing animal hosts or causing chronic infections have developed specific lysozyme evasion mechanisms. The most recently discovered mechanism is the production of specific lysozyme inhibitor proteins in gram-negative bacteria. The first such inhibitor (Ivy, *i*nhibitor of *v*ertebrate l*y*sozyme) was discovered fortuitously as a periplasmic *Escherichia coli* protein binding to and inhibiting with high affinity and specificity c-type lysozymes [5]. Since then, specific screens have resulted in the discovery of structurally different c-type lysozyme inhibitors as well as inhibitors that are specific for i- and g-type lysozymes [6]–[8], all from gram-negative bacteria. The newly discovered c-type inhibitor family comprises both periplasmic members (PliC, *p*eriplasmic *l*ysozyme *i*nhibitor of *c*-type lysozyme), and members that are bound to the luminal side of the outer membrane (MliC, *m*embrane bound *l*ysozyme *i*nhibitor of *c*-type lysozyme), while the i- and g-type inhibitors appear to be invariably periplasmic (PliI and PliG respectively). The number of inhibitor types (or gene homologs thereof) found in bacteria varies from none to all four. *E. coli*, which is the subject of the current work, produces active Ivy, MliC and PliG. By constructing knock-out mutants in various bacteria, all known inhibitors were shown to be at least partially protective against challenge with the corresponding type of lysozyme, and lysozyme inhibitors have therefore been proposed to play a role in host colonization by commensal or pathogenic bacteria [6]–[10]. In support of this hypothesis, Ivy was shown to be essential for the ability of *E. coli* to grow in human saliva and to enhance its ability to survive in egg white of chicken eggs, both of which contain only c-type lysozyme [10]. PliG, on the other hand, enhanced survival of *E. coli* in goose egg white, which contains only g-type lysozyme, but not in chicken egg white [11]. These results indicate that a highly specific one-to-one interaction between host lysozymes and bacterial lysozyme inhibitors may affect bacteria-host interactions. However, *in vivo* studies which demonstrate that lysozyme inhibitors affect the virulence of bacterial pathogens are still lacking to date. Therefore, the objective of this work was to investigate the role of lysozyme inhibitors in the virulence of *A*vian *P*athogenic E. c*oli* (APEC) in the chicken. APEC are a subset of extraintestinal pathogenic *E. coli* (ExPEC), besides uropathogenic *E. coli* (UPEC) and *E. coli* causing neonatal meningitis and septicemia (NMEC). In poultry, APEC are associated with extraintestinal infections, resulting in different diseases, of which colibacillosis, cellulitis and swollen head syndrome are the most predominant. Therefore, APEC is the cause of one of the most significant and widespread infectious diseases occurring in poultry and a cause of increased mortality and decreased economic productivity [12], [13]. A number of virulence factors of APEC have been established, including iron uptake systems [14], lipopolysaccharide O antigens and K1 capsule [15], fimbrial adhesins [16], autotransporter proteins [17] and a type VI secretion system [18], but the detailed mechanisms underlying pathogenicity are still poorly understood [19]. At the start of this study, all *E. coli* strains from which a genome sequence is available at NCBI (National Center for Biotechnology Information, http://www.ncbi.nlm.nih.gov), including APEC O1, contained a putative *ivy*, *mliC* and *pliG* gene. As such, APEC possesses the full complement of known inhibitors that can potentially interact with the c- and g-type lysozymes produced by the chicken. This match makes the APEC-chicken model well suited for the purpose of this work.

## Materials and Methods

### Bacterial strains and media

The bacteria and plasmids used in this work are described in Table 1. All the strains were grown in Luria-Bertani (LB) broth at 37°C. Antibiotics (Sigma-Aldrich, Bornem, Belgium) were added when appropriate at the following final concentrations: ampicillin (Ap), 100 µg/ml; kanamycin (Km), 50 µg/l; chloramphenicol (Cm), 20 µg/ml.

**Table 1:** Strains and plasmids.

Strain	Properties	Source
*E. coli* BL21		Novagen, Merck Biosciences, Darmstadt, Germany
APEC CH2	APEC strain CH2 is a virulent O78 *papG*II^+^ strain	[31], [32]
APEC CH2 Δ*ivy*::Km	*ivy* gene replaced by *aph* gene from pKD4; Km^R^	This study
APEC CH2 Δ*mliC*::Cm	*mliC* gene replaced by *cat* gene from pKD3; Cm^R^	This study
APEC CH2 Δ*ivy*::Km Δ*mliC*::Cm	APEC CH2 Δ*mliC*::Cm with Δ*ivy*::Km allele from *E. coli* TE2680; Km^R^, Cm^R^	This study, [9]
APEC CH2 Δ*pliG*::Cm	*pliG* gene replaced by *cat* gene from pKD3; Cm^R^	This study

Plasmid	Properties	Source
pKD3	Vector containing *cat* gene flanked by FRT sites, Cm^R^	[20]
pKD4	Vector containing *aph* gene flanked by FRT sites, Km^R^	[20]
pKD46	Plasmid expressing *γ*, *β* and *exo* recombination genes of phage λ under control of P_BAD_; temperature-sensitive replicon, Ap^R^	[20]
pSD19-Δ*ivy*::Km	Δ*ivy*::Km in pSD19, oriP1, Cm^R^	This study
pACYC177	Ap^R^;	New England Biolabs, Inc., Beverly, MA
pACYC-*ivy*	*ivy* under control of its own promotor in pACYC177; Ap^R^	This study
pACYC-*mliC*	*mliC* under control of its own promotor in pACYC177 Cm^R^, Ap^R^	This study
pACYC-*ivy/mliC*	*ivy* and *mliC* under control of their own promotor in pACYC177, Ap^R^	This study
pACYC-*pliG*	*pliG* under control of its own promotor in pACYC177 Cm^R^, Ap^R^	This study

### Construction of the APEC lysozyme inhibitor deletion and complemented strains

The deletion of *ivy*, *mliC*, *pliG* in APEC CH2 was achieved using the lambda red recombinase system described by Datsenko and Wanner [20] as adapted by Derbise *et al.* [21].

**Figure 1:**
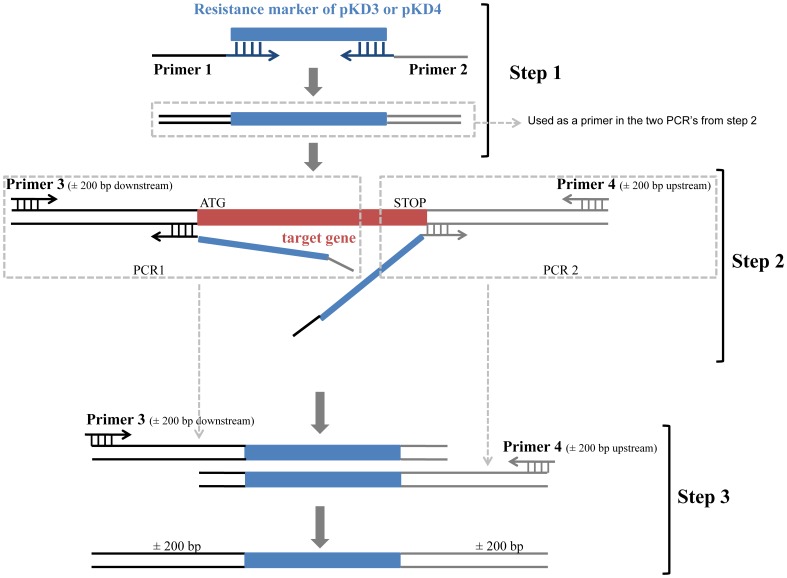
Scheme of three-step PCR to prepare DNA fragments for chromosomal gene replacement. In a first step, an antibiotic resistance cassette is amplified using primers carrying 5′ end 50 bp extensions homologous to the upstream (primer 2) and downstream (primer 1) region of the target inhibitor gene in APEC. The resulting PCR product is then used in a second step in combination with two other primers (primer 3 and primer 4) to separately amplify a larger part of the downstream and the upstream regions of the target gene. This results in two products which consist of the resistance marker cassette flanked by an upstream or a downstream 200 bp (or more) region homologous to the target gene. In a third step these two products are combined and amplified, resulting in a fusion product that has a large upstream and downstream homology region at either side of the resistance marker, and that is used for gene replacement.

A three-step PCR procedure (Figure 1) was used to produce an antibiotic resistance cassette flanked by long fragments homologous to the regions upstream and downstream of the gene to be replaced (*ivy*, *mliC* or *pliG*). In a first step the antibiotic resistance cassette from the plasmids pKD3 or pKD4 was amplified with Phusion DNA-polymerase (Finnzymes, Espoo, Finland) using 70 bp PCR primers comprising a 50 bp 5′ tail complementary to the region directly up- (primer 1) or downstream (primer 2) of the *E. coli* gene of interest. In a second step the APEC CH2 genomic DNA was used as a template in two separate PCR's to amplify ±200 bp fragments up- (primer 3) and downstream (primer 4) of the inhibitor gene. To this end, the PCR product obtained in the first step was used as a primer in combination with a new primer either 200 bases upstream or downstream of the gene. Finally, the products of the two PCR reactions from step 2 were combined in a third PCR step (without additional primers) to generate a PCR product comprising the antibiotic resistance cassette flanked by ±200 bp fragments corresponding to the upstream and downstream regions of the inhibitor gene. Gene disruption was carried out by electrotransformation of this final PCR product into APEC CH2 carrying the plasmid pKD46. Km or Cm resistant transformant colonies (depending on whether the Km resistance cassette from pKD4 or the Cm resistance cassette from pKD3 was used in the three-step PCR) were analysed by PCR (using primer 4 and a control primer) and DNA sequencing to confirm correct gene replacement. All the primers used in this procedure for each of the inhibitor genes are listed in Table S1. DNA sequencing was done by the Sanger method by the Division of Gene Technology, KU Leuven.

The resulting strains were designated APEC CH2 Δ*ivy*::Km, APEC CH2 Δ*mliC*::Cm and APEC CH2 Δ*pliG*::Cm.

The double knock-out strain APEC Δ*ivy*::Km Δ*mliC*::Cm was constructed by introducing the suicide plasmid pSD19 containing a Δ*ivy*::Km allele into APEC CH2 Δ*mliC*::Cm and selecting Km-resistant transformants. This Δ*ivy*::Km allele was obtained by PCR from *E. coli* TE2680, an earlier constructed *ivy* knock-out strain [9] with primers 5′-GATCTCTAGACGCCAGGCTTTAGGAGG-3′ and 5′-GATCAAGCTTCGGAGCCGAAAGGCTCC-3′, and the resulting blunt-end PCR product was ligated into pSD19 [22] cleaved with *Bam*HI. Correct gene replacement was verified by PCR and sequencing as described above.

For genetic complementation, all the inhibitor genes were cloned in the plasmid pACYC177. The APEC CH2 *pliG* gene with its own promotor was amplified with the following primers: 5′-GTGTAAGCTTGACATATAAATAGGTCTG-3′ and 5′-ATTAATCGATTTACTTAATTTGAATATC-3′. After digestion with *HindIII* and *ClaI* (recognition sites in primers are underlined), the resulting fragment was ligated into pACYC177 opened with the same restriction enzymes, and transformed into APEC CH2 Δ*pliG*::Cm. In a similar way, the APEC CH2 *mliC* gene and its promoter were amplified using the primers: 5′-ATTAATCGATACCCGTATTTCTCGCAA-3′ and 5′-TAGCGCTAGCCTTTACGGATTGTCAGTG-3′. After digestion with *ClaI* and *NheI*, the resulting fragment was ligated into pACYC177 cleaved with the same enzymes, and transformed into APEC CH2 Δ*mliC*::Cm. The two resulting plasmids were designated as pACYC-*pliG* and pACYC-*mliC*. Finally, the APEC CH2 *ivy* gene amplified with the primers 5′-ATATGGTACCGCCGCTGGTAAACATCTC-3′ and 5′-GATCAAGCTTGCGCTCCGTTTCTTTATCC-3′ was ligated into pACYC177 and pACYC-*mliC* opened with *SmaI*. The resulting plasmids pACYC-*ivy* and pACYC-*ivy/mliC* were respectively transformed into APEC CH2 Δ*ivy*::Km and APEC CH2 Δ*ivy*::Km Δ*mliC*::Cm.

### Isolation of periplasmic proteins

Periplasmic proteins were isolated by a cold osmotic shock procedure. In short, cells grown with shaking for 21 h in 50 ml LB broth at 37°C, harvested by centrifugation (10 min, 2900 g, room temperature), and resuspended in 6.25 ml 30 mM Tris-HCl (pH 8.0) with 20% (w/v) sucrose. After addition of 0.625 ml 10 mM EDTA (pH 8.0) and shaking for 10 min at room temperature, the sample was centrifuged (10 min, 6350 g, 4°C) and the cell pellet resuspended and shaken for 10 min in 6.25 ml ice-cold 5 mM MgSO_4_. This suspension was centrifuged again (10 min at 16800 g, 4°C), and the supernatant, corresponding to the periplasmic fraction, was stored at −20°C until further analysis.

### Lysozyme inhibition assay

Lysozyme inhibitory activity of bacterial periplasmic extracts was measured as described by Callewaert *et al.* [6] for c-type and by Vanderkelen *et al.* [8] for g-type lysozyme inhibitors, using respectively hen egg white lysozyme (HEWL; Fluka, 66000 U/mg protein) and recombinantly expressed g-type lysozyme from Atlantic salmon (SalG) [23].

### Serum resistance

For the serum resistance assay, all strains were grown 24 h in LB at 37°C. Blood was collected from healthy adult chickens and pooled. After clotting, normal chicken serum was isolated by centrifugation for 15 min at 3000 rpm. Inactivated chicken serum was prepared by incubating normal chicken serum in a waterbath at 56°C for 25 min to inactivate the complement system. Subsequently, the APEC strains and the serum-sensitive strain *E. coli* BL21 as a negative control were inoculated in the chicken serum at a concentration of approximately 10^5^ CFU/ml. Bacterial counts were determined immediately and after 3 h of incubation at 37°C by plating appropriate dilutions on LB plates.

### *In vivo* virulence test

Bacteria cultured for two subsequent periods of 24 h in LB at 37°C were diluted 1/100 in 4 ml fresh LB medium without antibiotics and grown until late exponential phase (OD_600 nm_ = 0.6, approximately 5×10^8^ CFU/ml). Part of this culture was diluted in LB to 5×10^7^ CFU/ml and 5×10^6^ CFU/ml. Colony counts were determined for each dilution by plating on LB agar to confirm these titers. Two hundred µL of each dilution was injected subcutaneously in the necks of 10 1-day-old broiler chicks (Ross Line, Belgabroed NV, Merksplas, Belgium), and mortality was monitored for 7 days. Control groups received 200 µL of sterile LB medium or 5×10^8^ CFU/ml of the non-pathogenic strain *E. coli* BL21. All experiments on animals were approved by the Ethical Commission for Experimental Use of Animals of the Katholieke Universiteit Leuven (Project number P116/2008).

### Statistical analysis

The statistical analyses of the *in vivo* experiments were performed using SAS software, version 8.2 (SAS Institute, Cary, NC, USA). Mortality rates for different strains with the same dosage were compared using the Kruskal-Wallis test.

## Results

### Construction of APEC CH2 inhibitor knock-out mutants

Since no genome sequence of APEC CH2 was available, we first confirmed by PCR and sequencing the presence of lysozyme inhibitor genes known to occur in other *E. coli* strains (*ivy*, *mliC* and *pliG*) in this strain (data not shown). To investigate the role of these lysozyme inhibitors in APEC virulence, we then constructed knock-outs of each of the genes, as well as a double *ivy* and *mliC* knock-out in order to have a strain producing neither c-type inhibitor. Each knock-out strain was also genetically complemented with a plasmid-borne copy of the corresponding gene(s), and the wild-type strain APEC CH2 was equipped with an empty pACYC177 plasmid to detect any potential influence of the presence of the plasmid. The successful construction of the inhibitor knock-out mutants as well as the plasmids was confirmed by PCR and sequencing. Subsequently, periplasmic extracts of all the strains were analyzed for inhibitory activity against c- and g-type lysozyme (Table 2). Since MliC is an outer membrane protein, we also attempted to measure the loss of inhibitory activity in the membrane fraction of the *mliC* mutant, but no activity exceeding the noise level could be detected even in the wild-type strain. This was not unexpected, because we had observed previously that also *E. coli* MG1655 does not produce detectable MliC levels, and because *mliC* is poorly expressed under normal laboratory growth conditions [6]. All knock-outs caused a considerable reduction of the periplasmic lysozyme inhibitory activity, but the residual activities varied. Knock-out of *pliG* reduced g-type inhibitory activity in the periplasmic extract to a background level, while the c-type inhibitory activity was only partly reduced in the *ivy* knock-out. The latter was unexpected, because Ivy is the only known periplasmic lysozyme inhibitor in *E. coli*, and knock-out of *ivy* completely eliminated c-type lysozyme inhibitory activity in periplasmic extracts of *E. coli* MG1655 [9]. Finally, double knock-out of *ivy* and *mliC* also reduced c-type inhibitory activity in the periplasmic extracts, but somewhat less than the individual *ivy* knock-out. Complementation of each knock-out strain with the corresponding gene or genes increased the inhibitory activity back to the wild-type level. All the constructed knock-out mutants and genetically complemented mutants showed growth curves in LB broth at 37°C that were undistinguishable from the parental APEC CH2 growth curve (data not shown).

**Table 2:** Inhibitory activity of periplasmic extracts of different APEC strains.

Strain	Inhibitory activity^a^ (IU/ml) against HEWL	Inhibitory activity* (IU/ml) against SalG
APEC CH2	34.2±2.7	18.1±2.0
APEC CH2 Δ*ivy*::Km	8.3±1.7^a^	nd
APEC CH2 Δ*ivy*::Km pACYC-*ivy*	34.7±2.3	nd
APEC CH2 Δ*mliC*::Cm	8.0±3.6^a^	nd
APEC CH2 Δ*mliC*::Cm pACYC-*mliC*	18.5±10.0	nd
APEC CH2 Δ*ivy*::Km Δ*mliC*::Cm	14.6±1.5^a^	nd
APEC CH2 Δ*ivy::*Km Δ*mliC*::Cm pACYC-*ivy/mliC*	34.8±1.8	nd
APEC CH2 Δ*pliG*::Cm	nd	2.5±0.5^a^
APEC CH2 Δ*pliG*::Cm pACYC-*pliG*	nd	23.4±6.3

* *Experiments were performed in triplicate and mean inhibitory activity (IU/ml) and standard deviation are shown. Since* MliC *is a membrane protein, its activity can not be measured in a periplasmic extract.*

a *Inhibitory activity differing significantly (p<0.05) from that of the wild-type strain in the same column.*

### Serum sensitivity

As a first approach to assess the virulence of the different APEC inhibitor knock-outs, a serum sensitivity test was conducted. For many pathogens including APEC, the ability of bacteria to survive and grow in blood serum is a prerequisite for virulence and has proven useful in discriminating virulent and avirulent isolates [24]. Serum resistance depends on the ability of the bacteria to overcome the combined antibacterial effectors that are present in serum. One of the most powerful effectors is the multimolecular attack complex formed by the complement system [25], but there is also a contribution of several antibacterial peptides and proteins with specific modes of action, such as lysozyme. The results of the serum resistance tests are shown in Table 3. All strains including *E. coli* BL21, which was included as a serum-sensitive control, showed strong growth in serum that had been heat-treated to inactivate the complement system (plate counts increasing 250- to 350-fold after 3 h, no significant differences). In untreated serum, growth of BL21 and APEC CH2 was reduced to 13.8% and 60.2% of the growth level in heat-treated serum, respectively, indicating that the avirulent and virulent controls could be distinguished in the serum test (p<0.05). For three APEC mutants, the reduction in growth in untreated serum was in the same range as for the parent strain (60–70%), but for the *mliC* mutant, a reduction to the level of BL21 was observed (10.3%). This result implicates that the *mliC* knock-out strain could have a reduced virulence whereas the *ivy mliC* knock-out strain and the other inhibitor knock-out strains are considered to be as virulent as the wild-type APEC strain. To confirm these observations, the virulence of the strains was analyzed *in vivo*.

**Table 3:** Serum resistance of different APEC strains.

Strain	Relative growth^a^ in serum
*E. coli* BL21	13.8±9.5%*
APEC CH2	60.2±2.1%
APEC CH2 Δ*ivy*::Km	60.2±4.3%
APEC CH2 Δ*mliC*::Cm	10.3±2.9%*
APEC CH2 Δ*ivy*::Km Δ*mliC*::Cm	64.5±6.0%
APEC CH2 Δ*pliG*::Cm	71.6±29.1%

a *Relative growth is the increase in plate count (N_3 h_/N_0 h_) in serum expressed relative to the increase in plate count in heat-inactivated serum ( = 100%). N_3 h_/N_0 h_ ranged between 240 and 347 in heat-inactivated serum. Mean values+standard deviations for three independent cultures are shown. Significant differences (p<0.05) with the wildtype APEC CH2 strain are indicated with an asterisk.*

### In vivo virulence

**Figure 2:**
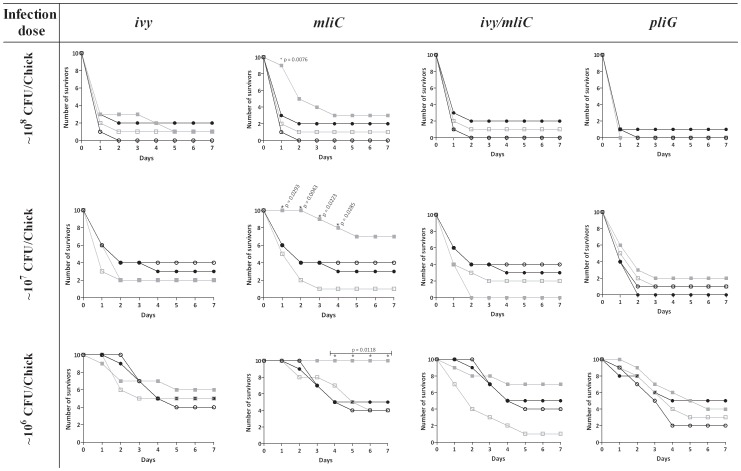
Mortality curves of 1-day old chickens upon subcutaneous infection with APEC strains. Number of surviving animals up to 7 days post infection with APEC CH2 (•), APEC CH2 pACYC177 (empty plasmid control) (○), APEC inhibitor knock-out (▪) and the corresponding complemented APEC inhibitor knock-out strain (□). Time points where the number of survivors with the inhibitor knock-out was significantly different from that with the wild-type are marked with ‘*’ and the corresponding p-value.

A first *in vivo* experiment was conducted with the *ivy*, *mliC* and *ivy mliC* mutants to investigate the role of the c-type lysozyme inhibitors in virulence. The mortality curves up to 7 days post infection are shown in Figure 2. The laboratory strain *E. coli* BL21, included as a negative control, was confirmed to be non-virulent since no chicks died even with the highest dose of 10^8^ CFU per animal (data not shown). On the other hand, the wild-type CH2 strain caused a dose-dependent mortality, and even at the lowest dose of 10^6^ CFU/animal killed 6 out of 10 chicks after 7 days. Interestingly, virulence was clearly reduced by knock-out of MliC as anticipated by the outcome of the serum resistance test. At a dose of 10^8^ CFU/animal the onset of mortality for the *mliC* mutant showed a statistically significant delay of one day compared to the wild-type (p = 0.0076), and this mutant killed less animals after 7 days compared to the wild-type strain and the complemented *mliC* mutant, although this difference was not significant. However, at lower doses the reduced mortality with the *mliC* mutant became more pronounced. At 10^7^ CFU/animal, the difference with the wild-type strain was significant from day 1 to day 4, while at 10^6^ CFU/animal it was significant from day 4 until the end of the observation period (Figure 2). At this dose, the chicks infected with the *mliC* mutant showed 100% survival compared to only 50% for the wild-type strain (p = 0.0118). Complementation of the knock-out with a plasmid-borne *mliC* gene reverted the mortality rate to wild-type level (50%). With respect to complementation, the control experiment with the wild-type strain containing the empty cloning vector pACYC177 indicated the absence of any significant effect of the plasmid on the mortality rates. A more quantitative analysis was performed by calculating LD_50_ values from the data of this experiment according to the method of Reed and Muench [26], and this revealed an at least thirtyfold increased LD_50_ for the *mliC* knock-out strain (3.2×10^7^ CFU/chick) compared to the wild-type (1.0×10^6^ CFU/chick). This *in vivo* experiment was repeated, resulting in similar mortality curves (Figure S1) and identical LD_50_ values. As opposed to *mliC*, *ivy* had no major detectable effect on virulence in both infection experiments. However, similar as in the serum test, the *ivy mliC* double knock-out caused higher mortalities than the *mliC* mutant at a dose of 10^7^ CFU/ml (p = 0.0014) and at a dose of 10^6^ CFU/ml (p = 0.0671). At the lowest infection dose of 10^6^ CFU/ml the mortality caused by *mliC* differs significantly from the mortality caused by all the other strains (p<0.05) except for the double knock-out strain (p = 0.0671).

A separate challenge experiment was conducted with the *pliG* knock-out strain, its complemented derivative and the wild-type strain, but no significant differences in mortality rates were observed at any of the three applied doses (Figure 2).

## Discussion

In this work we investigated the role of lysozyme inhibitors in bacterial virulence using an APEC – chicken model system. Single knock-outs of *ivy*, *mliC* and *pliG* as well as an *ivy*/*mliC* double knock-out were successfully constructed in APEC CH2, and plasmid-based complementation of the mutants with the corresponding genes was accomplished. First we determined the serum resistance of the mutants as a rapid and simple indicator of virulence, and found that *mliC*, but not *ivy* or *pliG*, was required for serum resistance of APEC CH2. Although bacterial sensitivity to serum is mainly due to the action of the complement system, there is also a contribution of other antimicrobial components such as lysozyme. The action of the membrane attack complex of the complement system destabilizes the outer membrane and may render it permeable to lysozyme. Conversely, degradation of the peptidoglycan layer may facilitate pore formation in the cytoplasmic membrane by the membrane attack complex, resulting in cell leakage an death [27]. Our results suggest that MliC can neutralize this contribution of serum lysozyme to complement activity. Given the effect of the single knock-outs, the parental level of serum resistance in the *mliC ivy* double knock-out was unexpected (see also below).

Subsequently, infection experiments with 1-day old chickens subcutaneously injected with different doses of bacteria (10^6^, 10^7^ and 10^8^ CFU/chick) confirmed the attenuated virulence of the *mliC* mutant. In addition, virulence was fully restored by complementation with the *mliC* gene. As anticipated from the serum resistance test, *pliG* nor *ivy* had any significant effect on virulence. Since PliG is the only known inhibitor of g-type lysozyme in APEC, and its knock-out reduced g-type lysozyme inhibitory activity of APEC CH2 to background levels, it can be concluded that PliG is not required for virulence of this pathogen, at least not in the subcutaneous infection model used in this work. Of course, a role of this inhibitor in other commensal or pathogenic bacteria – host interactions can not be excluded on the basis of these observations. For the c-type lysozyme inhibitors, the situation is more complex. Based on the observations with the single knock-out strains, the outer membrane-bound inhibitor MliC appears to play a role in virulence, but not the periplasmic inhibitor Ivy. Since MliC is an outer membrane protein, there could be some concern that knock-out of MliC could have destabilized the outer membrane, thus rendering the bacteria more sensitive to a variety of antibacterial effectors from its host. This appears not to be the case, because the *mliC* mutant retained its resistance to detergents when plated on LB containing 2.0% SDS or 2.0% Triton X-100 (data not shown), whereas mutants with outer membrane defects typically display a high serum and detergent sensitivity [28], [29]. Therefore, we can have confidence that the attenuated virulence of the *mliC* mutant is genuinely linked to its reduced production of c-type lysozyme inhibitor rather than to an indirect effect. One point that needs further clarification is which inhibitor is responsible for the attenuated virulence, since the *mliC* mutant unexpectedly showed a considerably reduced level of periplasmic lysozyme inhibitor activity (Table 2).

An additional complication, in line with the observations in the serum resistance test, is that introduction of an *ivy* knock-out into the *mliC* mutant restored the attenuated virulence of the latter to almost wild-type level again, indicating that there is some type of interference between these two mutations. Comparison of the periplasmic lysozyme inhibitory activities confirms that this is indeed the case, because the level in the double mutant (14.6 IU/ml) is higher than that in the *mliC* mutant (8.0 IU/ml). For comparison, an *ivy mliC* mutant of *E. coli* MG1655 was previously shown to have no residual periplasmic lysozyme inhibitory activity [6], but an explanation for this strain-dependent behaviour is currently lacking. However, we found that two *E. coli* genome sequences that were added to the NCBI genome database during the preparation of our manuscript contain a *pliC* homolog in addition to *ivy* and *mliC*, unlike all other *E. coli* genomes. This is not the case for the APEC O1 genome, but nevertheless, the residual periplasmic lysozyme inhibitory activity of the APEC CH2 *ivy* knock-out could indicate that this strain also has an additional *pliC*.

In conclusion, this work is the first to demonstrate the involvement of a lysozyme inhibitor in bacterial virulence. Although findings from the APEC – chicken model system studied in this work cannot be simply extrapolated to other pathogen – host interactions, the wide distribution of different types of lysozyme inhibitors in bacteria suggests that these molecules have evolved as virulence factors or effectors of commensal interactions in a wide range of bacteria. This finding may also open perspectives for new avenues for the development of antibacterial drugs, for example by designing compounds that can neutralize bacterial lysozyme inhibitors, thus rendering them more sensitive to the host lysozymes [30].

## Supporting Information

Figure S1 Mortality curves of 1-day old chickens upon subcutaneous infection with APEC strains (repeat experiment). Number of surviving animals up to 7 days post infection with APEC CH2 (•), APEC CH2 pACYC177 (empty plasmid control) (○), APEC inhibitor knock-out (▪) and the corresponding complemented APEC inhibitor knock-out strain (□). Time points where the number of survivors with the inhibitor knock-out was significantly different from that with the wild-type are marked with ‘*’ and the corresponding p-value.
(TIF)

Table S1 Oligonucleotide primers used for construction and verification of the APEC inhibitor knock-out mutants by three-step PCR procedure. Primer numbering from 1 to 4 for each inhibitor corresponds with numbering in Figure 1, which explains construction of the gene replacement cassettes by three-step PCR procedure.
(DOCX)
