# Supplementary figures and images for: Role of Lysozyme Inhibitors in the Virulence of Avian Pathogenic Escherichia coli
Source: PLoS One. 2012 Sep 26;7(9):e45954. doi: 10.1371/journal.pone.0045954 (PMC3458809; doi:10.1371/journal.pone.0045954)

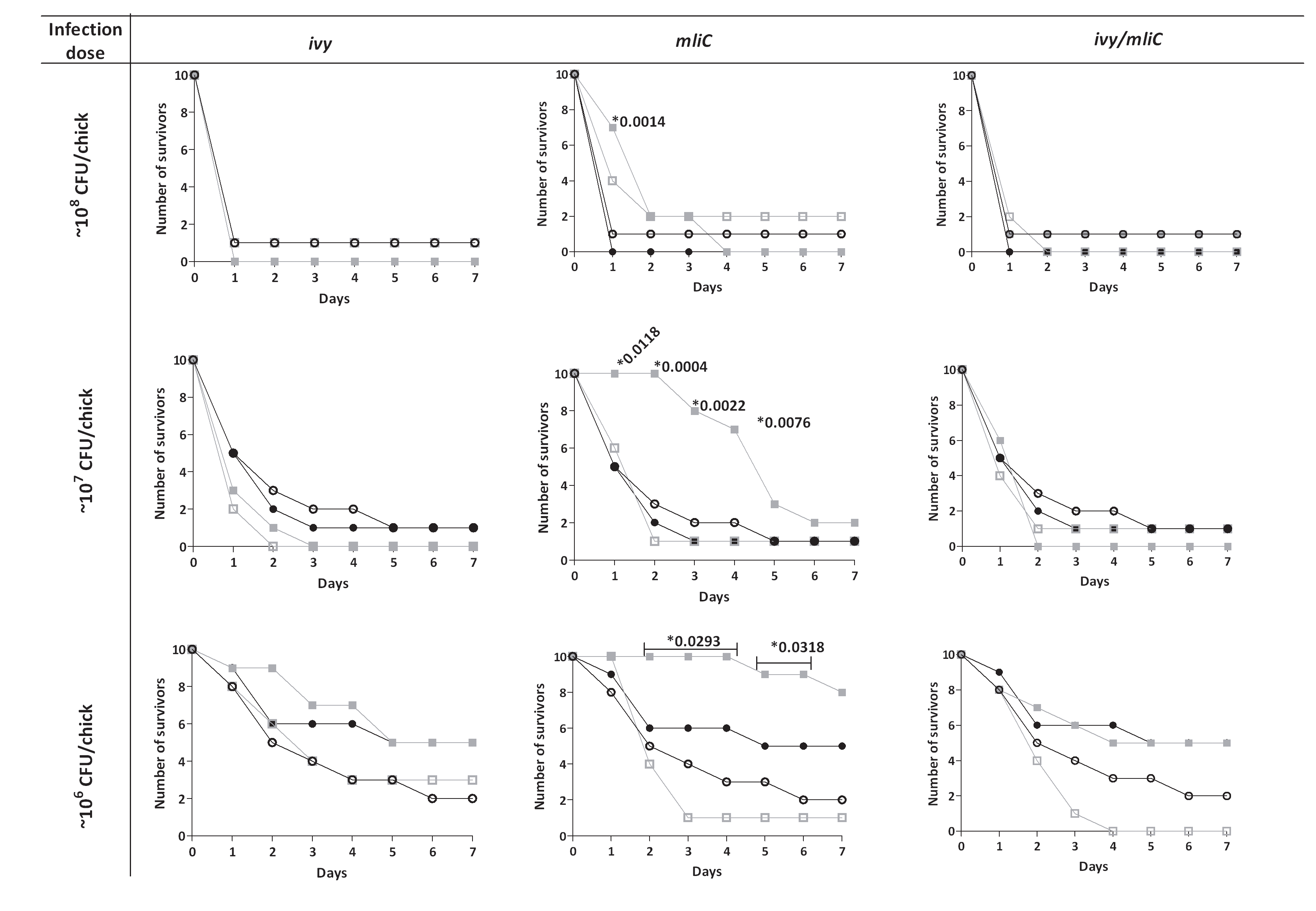

Supplement: Figure S1 — Mortality curves of 1-day old chickens upon subcutaneous infection with APEC strains (repeat experiment). Number of surviving animals up to 7 days post infection with APEC CH2 (•), APEC CH2 pACYC177 (empty plasmid control) (○), APEC inhibitor knock-out (▪) and the corresponding complemented APEC inhibitor knock-out strain (□). Time points where the number of survivors with the inhibitor knock-out was significantly different from that with the wild-type are marked with ‘*’ and the corresponding p-value. (TIF) [file pone.0045954.s001.tif]
