# Supplementary material for: Role of Lysozyme Inhibitors in the Virulence of Avian Pathogenic Escherichia coli
Source: PLoS One. 2012 Sep 26;7(9):e45954. doi: 10.1371/journal.pone.0045954 (PMC3458809; doi:10.1371/journal.pone.0045954)
Supplement: Table S1 — Oligonucleotide primers used for construction and verification of the APEC inhibitor knock-out mutants by three-step PCR procedure. Primer numbering from 1 to 4 for each inhibitor corresponds with numbering in Figure 1, which explains construction of the gene replacement cassettes by three-step PCR procedure. (DOCX) [file pone.0045954.s002.docx]

| Primer Name | Sequence |
| --- | --- |
| primer1_ivy | 5’-TCTGATTTGCAAATTATCGTGTTATCGCCAGGCTTTA GGAGGTTAATAACGTGTAGGCTGGAGCTGCTTC-3’ |
| primer2_ivy | 5’-CCGCGTAAATGAATAACGGAGCCGAAAGGCTCCGTT TCTTTATCCGCTAAATGGGAATTAGCCATGGTCC -3’ |
| primer3_ivy | 5’-GTAGGGATCCGAGATTGCGCGACCACACAGG-3’ |
| primer4_ivy | 5’-GAAGGGGCTGATTGATAAAG-3’ |
| control_ivy | 5’-ACCTAAACTCATCCGCATCC-3’ |
| primer1_mliC | 5’-GAACGTTAGGGAGGGCGTATTGCCCTCCAGACCAGG AAAGTCTTCGGGATGTGTAGGCTGGAGCTGCTTC-3’ |
| primer2_mliC | 5’-TTACGGATTGTCAGTGGGTGACGCTATTGTGCGCCG CCCCTGGAAAAATCATGGGAATTAGCCATGGTCC-3’ |
| primer3_mliC | 5’-GCTATTTTCCCCGCTAACC-3’ |
| primer4_mliC | 5’-ACAAGCCTGAGAGAGCCAAC-3’ |
| control_mliC | 5’-ATTAGGATCCACCCGTATTTCTCGCAA-3’ |
| primer1_pliG | 5’-TTGTTATATTATAACAGTTCATCGTACTCATTCTGAA CAGGAGACTACCAGTGTAGGCTGGAGCTGCTTC-3’ |
| primer2_pliG | 5’-GGAGTGGGGCATTAACGTTTATGACGGGGAGAGTCC CCGTCGGTTGACATATGGGAATTAGCCATGGTCC-3’ |
| primer3_pliG | 5’-GTGTGGATCCGACATATAAATAGGTCTG-3’ |
| primer4_pliG | 5’-CAATCAAACCACCACAAAGA-3’ |
| control_pliG | 5’-TAACATCAATGAAACCCGC-3’ |
